# Higher Dimensional Gaussian-Type Solitons of Nonlinear Schrödinger Equation with Cubic and Power-Law Nonlinearities in PT-Symmetric Potentials

**DOI:** 10.1371/journal.pone.0115935

**Published:** 2014-12-26

**Authors:** Yi-Xiang Chen, Fang-Qian Xu

**Affiliations:** School of Electronics Information, Zhejiang University of Media and Communications, Hangzhou, 310018, P.R.China; Shanxi University, China

## Abstract

Two families of Gaussian-type soliton solutions of the (n+1)-dimensional Schrödinger equation with cubic and power-law nonlinearities in 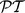-symmetric potentials are analytically derived. As an example, we discuss some dynamical behaviors of two dimensional soliton solutions. Their phase switches, powers and transverse power-flow densities are discussed. Results imply that the powers flow and exchange from the gain toward the loss regions in the 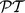 cell. Moreover, the linear stability analysis and the direct numerical simulation are carried out, which indicates that spatial Gaussian-type soliton solutions are stable below some thresholds for the imaginary part of 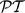-symmetric potentials in the defocusing cubic and focusing power-law nonlinear medium, while they are always unstable for all parameters in other media.

## Introduction

The construction of the exact solutions of nonlinear partial differential equations is one of the most important and essential tasks in various branches from mathematical physics, engineering sciences, chemistry to biology [1], [2]. Many powerful methods have been presented, such as the (G'/G)-expansion method [3]–[5], the variable separation method [6], [7], the multiplier approach [8], the similarity transformation method [9] and the extended generalized Riccati equation mapping method [10], and so on.

The nonlinear Schrödinger equation (NLSE) and its relatives play important role in in physics, biology and other fields. Researchers have studied abundant mathematical solutions and physical localized structures of various NLSEs, including solitons and nonautonomous solitons [11], [12], similaritons [13], rogue waves [14] and breathers [15] etc.

In recent years, the propagation of solitons in parity-time (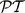) symmetric potentials are presently attracting a great interest both from the theoretical and from the applicative point of view [16]–[26]. The definitions of 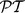 potentials were given by Bender and coworkers in classical quantum mechanics, namely, the 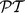-symmetric potential satisfies 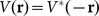 with 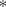 denoting complex conjugation [27]. Considering the mathematical correspondence between the quantum Schrödinger equation and the paraxial equation of diffraction-NLSE, the concept of 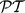 symmetry has been introduced in the field of optics. Pioneering theoretical contributions of Christodoulides and co-workers [16], [17] stimulated recent experimental observations [18], [19]. After then, optical solitons in 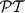-symmetric Rosen-Morse potential [20], periodic potential [21] and Scarf II potential [22] were discussed. Two-dimensional (2D) solitons in nonlocal media [23] with 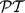-symmetric potentials have also been reported. More recently, the propagation of nonautonomous solitons in optical media with 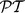 symmetry has been a subject of intense investigation [24]–[26].

However, all soliton solutions in [20]–[26] are sech-type. Other type of soliton solutions in 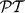-symmetric potentials is less studied. Especially, higher dimensional Gaussian-type soliton solutions in 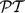-symmetric potentials with cubic and power-law nonlinearities are hardly studied. In this paper, we aim to obtaining some analytical higher dimensional Gaussian-type soliton solutions of NLSE with cubic and power-law nonlinearities in 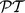-symmetric potentials. Two issues are firstly studied in this present paper: i) higher dimensional Gaussian-type soliton solutions are analytically presented in 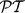-symmetric media with cubic and power-law nonlinearities, and ii) linear stability analysis and direct simulation are used together to investigate the stability of solutions in 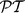-symmetric media with cubic and power-law nonlinearities.

## Results

### Analytical higher dimensional Gaussian-type soliton solutions

The propagation of spatial soliton and LB in a 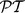-symmetric nonlinear medium of non-Kerr index can be described by the following NLSE
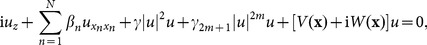
(1)where 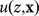 with 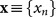 is the complex envelope of the electrical field, 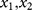 and 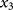 represent the transverse spatial coordinates 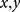 and the retarded time 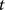, respectively. The subscripts 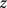 and 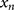 in the first and second terms in Eq. (1) denote the derivatives to them. Parameters 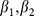 and 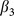 are respectively the coefficients of the diffraction and dispersion, 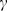 is the cubic nonlinear coefficient and 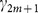 for 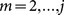 describe the nonlinearities of orders up to 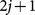. For 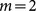 one has the quintic nonlinearity, for 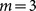 the septic, and so on. Functions 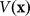 and 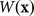 describe the index guiding and the gain/loss distribution respectively. The real and imaginary components of the complex 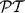-symmetric potential satisfy 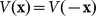 and 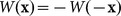. If 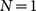, Eq. (1) is 1DNLSE, and its solutions are 1D spatial soliton solutions. If 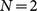, Eq. (1) is 2DNLSE, and its solutions are 2D spatial soliton solutions. If 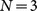, Eq. (1) is 3DNLSE, and its solutions are LB solutions.

We seek solutions of NLSE (1) in the form:
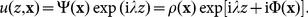
(2)


Substituting it into Eq. (1) leads to two differential equations about real functions 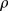 and 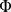


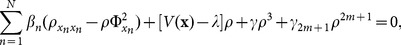
(3)

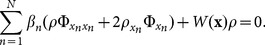
(4)


In the following, we derive analytical Gaussian-type soliton solutions of Eqs. (3) and (4) in two kinds of 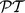-symmetric potentials.

**Family 1 Solution in the first type of extended** 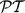 **-symmetric potential.** If the 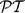-symmetric potential has the form
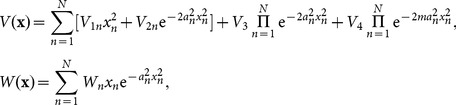
(5)with real parameters 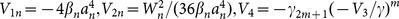 and arbitrary constants 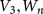 and 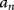, the localization condition 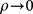 as 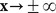 brings into solution of Eq. (1) as follows
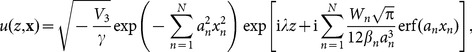
(6)with 
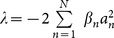
 and the error function 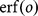.

**Family 2 Solution in the second type of extended** 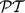 **-symmetric potential.** When the 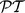-symmetric potential has the form
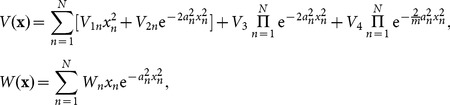
(7)with real parameters 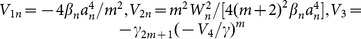 and arbitrary constants 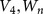 and 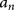, the localization condition 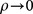 as 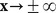 leads to solution of Eq. (1) in the form
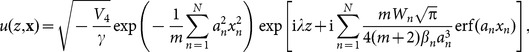
(8)with 
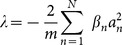
 and the error function 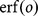.

If 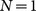, solutions (6) and (8) are 1D spatial soliton solutions with 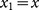. If 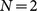, solutions (6) and (8) are 2D spatial soliton solutions with 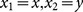. If 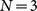, solutions (6) and (8) are LB solutions with 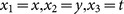. From solutions (6) and (8), one knows that 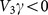 or 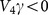, thus solution (6) and (8) exist in self-focusing cubic (FC) media with positive cubic nonlinearity (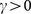) if 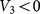 or 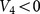, as well as in self-defocusing cubic (DC) media with negative cubic nonlinearity (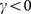) if 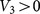 or 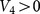.

### Characteristic quantities of analytical solutions

**Figure 1:**
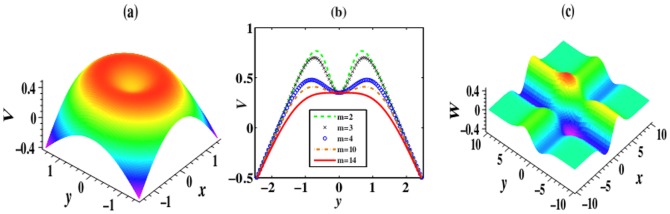
All pictures are the cases of 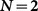. (a) and (c) 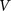 and 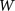 in the 2D 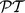-symmetric potential expressed by (5) with 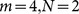. (b) The comparison of 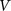 for different 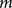 at 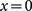. Parameters are chosen as 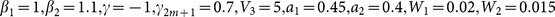.

It is obvious that the real and imaginary parts of the 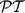-symmetric potentials (5) and (7) are both even and odd functions with regards to 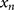. Thus, 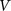 and 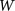 exhibit the symmetric and anti-symmetric properties. As an example, we present 2D case of these properties in Fig. 1. From Fig. 1(b), 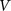 possesses the plateau-like structure with 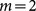. with the increase of 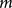, this structure turns into a two-hump structure, and the humps protrude little by little.

**Figure 2:**
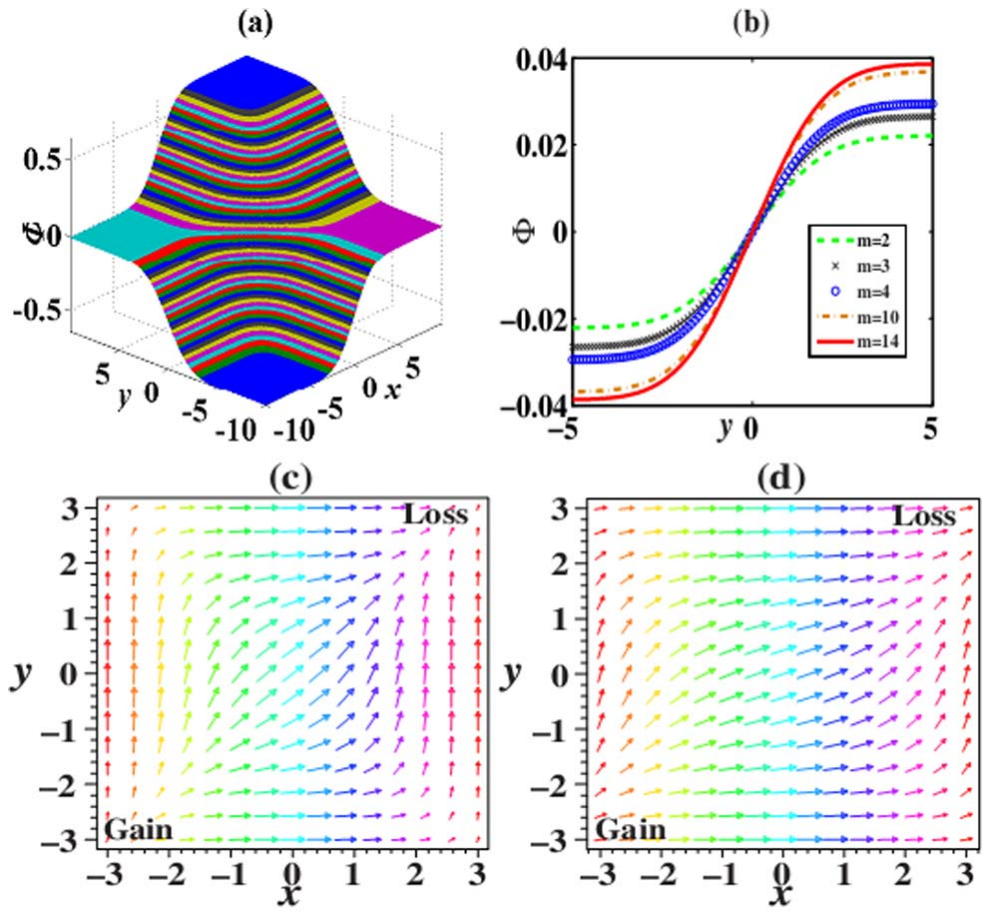
All pictures are the cases of 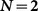. (a) Phase switch of solution (6), (b) the comparison of phase switch of solution (8) with the different 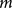 at 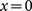. (c) and (d) Power-flow vector 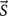 for solutions (6) and (8) with 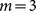. Parameters are chosen as 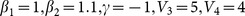 with (a),(c) 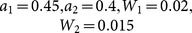 and (b),(d) 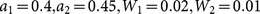.

In the 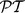-symmetric potentials above, the phase switches of solutions (6) and (8) can be found. Fig. 2(a) presents the phase switch of solution (6). The comparison of phase switch of solution (8) with the different 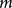 at 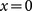 is shown in Fig. 2(b). The span of switch gradually enlarges with the increasing order 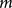 of nonlinearity.

The power 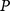 and power-flow density (Poynting vector) 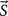 are two important quantities. They can be calculated from 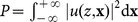, and 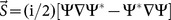. From these definitions, the powers 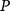 of solution (6) and (8) can be expressed 
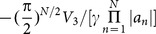
 and 
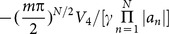
, respectively. The power-flow densities 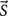 of solution (6) and (8) can be expressed as 
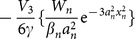
 and 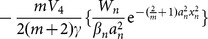, respectively. Note that the notation 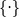 combines multi-component case, which means that there is one component for 1D case, and two components 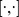 for 2D case, etc.

From these detailed expression above, We can find that the power and the power-flow density of solution (6) are both independent of the parameter 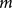, while those of solution (8) both depend on the parameter 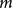. Considering 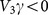 or 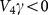, 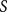 is everywhere positive, which implies that the power flow and exchange for solutions (6) and (8) in the 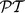 cells are always in one direction, that is, from the gain toward the loss regions. Two examples to this exchange among gain or loss regions are shown in Fig. 2(c) and 2(d) for 2D case.

When 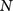 is chosen as other values, such as 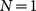 and 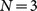, the phase switch and the directional power-flow density can also be found. Here we omit these discussions for the limit of the length. In the next section, we only focus on the dynamical behaviors of solutions (6) and (8) with 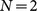.

## Discussion and Analysis

### Linear stability analysis of analytical solutions

In order to discuss the linear stability of analytical solutions (6) and (8) of Eq. (1), we consider a perturbed solution [28] 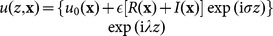, where 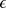 is an infinitesimal amplitude, 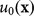 is a respective eigenmode [analytical solution of Eq. (1)], 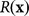 and 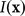 are the real and imaginary parts of perturbation eigenfunctions, which may grow upon propagation with the perturbation growth rate 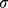. It is obvious that the perturbed solution becomes linearly unstable if there exist nonzero imaginary parts of 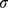, otherwise solution is stable.

Substituting the perturbed solution into Eq. (1) and linearizing it around the unperturbed one (the first-order term of 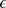), we arrive at the eigenvalue problem
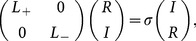
(9)where 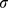 is an eigenvalue, 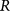 and 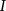 are eigenfunctions with Hermitian operators 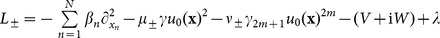 with 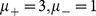 and 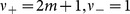.

In the following, we discuss the whole eigenvalue spectra of the above problem (9). In order to perform the numerical computation, here we have restricted 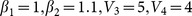 with 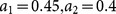 in solution (6) and 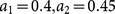 in solution (8) with 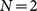. We discuss the linear stability analysis of solutions (6) and (8) in the self-focusing and self-defocusing cubic and power-law nonlinearities.

**Figure 3:**
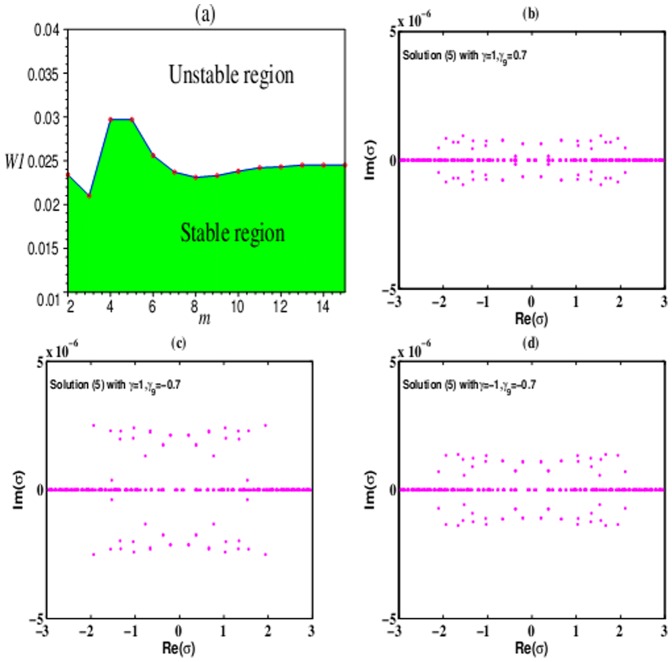
All pictures are the cases of 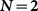. (a) Stable and unstable regions of solution (6) with some order parameters 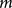 for the DC and FP nonlinearities. Eigenvalues of solution (6) for (b) the FC and FP nonlinearities, (c) the FC and DP nonlinearities and (d) the DC and DP nonlinearities. Parameters are chosen as 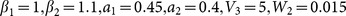 with (a) 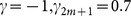, (b)-(d) 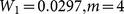.

Fig. 3(a) presents stable and unstable regions of some order parameters 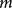 for the self-defocusing cubic (DC) and self-focusing power-law (FP) nonlinearities in 2D case when other parameters are chosen as 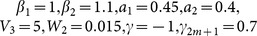. For a certain 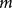, if other parameters are fixed, there exists a threshold value of 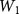, above which analytical solution becomes unstable and below which analytical solution evolves stably. From Fig. 3(a), the threshold value of 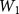 decreases at first, next adds to a maximum when 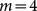, then attenuates again, and finally increases to a certain value when 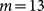. For 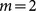, the threshold value of 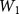 is close to 0.0234. For 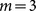, it is 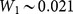, and for 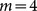, 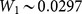. For 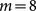, 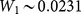. After 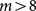 the threshold value of 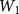 increases, and reaches to a fixed value (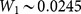) after 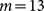.

For other nonlinearities such as the self-focusing cubic (FC) and self-focusing power-law (FP) nonlinearities, the self-focusing cubic (FC) and self-defocusing power-law (DP) nonlinearities and the self-defocusing cubic (DC) and self-defocusing power-law (DP) nonlinearities, the eigenvalue 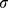 of solution (6) exists many imaginary parts shown in Fig. 3(b)-3(d), and thus solution (6) is always unstable in these nonlinear media.

The linear stability analysis of solution (8) has the similar result with that of solution (6). In the FC and FP, the FC and DP, and the DC and DP nonlinear media, the eigenvalue 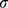 of solution (8) appears many imaginary parts, and thus solution (8) is also linearly unstable. In the DC and FP nonlinear medium, if all other parameters are fixed, solution (8) is linearly stable only in the case when the values of 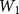 and 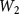 are chosen below their threshold values. Table 1 lists the thresholds of 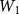 and 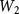 of solution (8) for 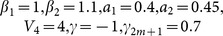 in 2D case with 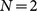.

**Table 1:** The thresholds of *W*_1_ and *W*_2_ for solution (7).

m	*W*_1_	*W*_2_
**2**	0.0031	0.001
**3**	0.003	0.001
**4**	0.0022	0.001
**5**	0.00011	0.00014
**6**	0.000076	0.00011

Table 1 indicates that the values of 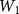 and 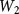 decrease quickly with the increase of the order parameter 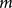. Different from the case in Fig. 3(a), the values of 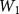 and 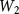 always decrease in Table 1. The gain (loss) related to the values of 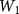 and 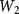 are fairly small compared with the bigger value of 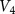, otherwise, all analytical solutions finally result in instability.

### Numerical calculation for the stability of analytical solutions

The linear stability analysis gives the stable regions of analytical solutions in different 2D extended 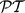-symmetric potentials. However, the analytical solutions are not exactly satisfied in real situations, thus it is important to discuss the stability of solutions with respect to finite perturbations. In the following, we further discuss the stability of these solutions against a perturbation of 5% white noise by the direct numerical simulation (a split-step Fourier beam technique).

**Figure 4:**
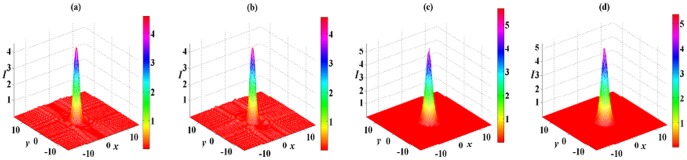
Initial value of solution (6) with 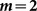 in the 2D extended 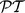-symmetric potential (5) at 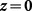 in (a). The numerical reruns of solution (6) with (b) 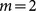, (c) 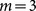 and (d) 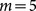 for DC and FP nonlinearities at 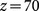. A 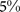 white noise are added to the initial values. All parameters are chosen as the same as those in Fig. 3 except for 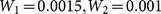.

**Figure 5:**
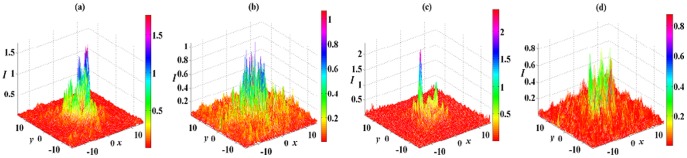
Unstable 2D spatial soliton solution (6) in the 2D extended 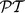-symmetric potential (5). The numerical reruns of solution (6) with (a),(c) 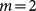, (b),(d) 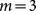 for (a), (b) DC and DP nonlinearities with 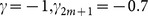 and (c), (d) FC and DP nonlinearities with 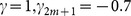 at 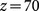. All other parameters are chosen as the same as those in Fig. 4.

The stable and unstable 2D spatial soliton solution (6) in the 2D extended 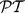-symmetric potential (5) are presented in Figs. 4 and 5, respectively. From Fig. 4, 2D spatial soliton solution (6) with 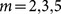 stably propagate over tens of diffraction lengths in the DC and FP nonlinear medium, and do not yield any visible instability except for some small oscillations. We can find a good agreement with results from the linear stability analysis for analytical solution (6). This indicates that the 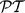 complex potential is strong enough to inhibit the collapse of spatial soliton solutions caused by diffraction and DC and FP nonlinearities. From Fig. 4(b), when 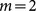, the white noise only influences the background of soliton and produces some small oscillations around the soliton. When 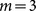 and 5, the white noise brings some small oscillations on the top part of soliton shown in Fig. 4(c) and 4(d).

In other nonlinear media, solution (6) is unstable in the 2D extended 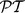-symmetric potential (5). As two examples, Fig. 5 displays this kind of instability. For the DC and DP nonlinearities and the FC and DP nonlinearities, these solitons can not maintain their original shapes. Along the propagation distance, these solitons collapse, and finally decay into noise. With the increase of nonlinear order parameter 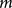, the instability of solution (6) adds. Compared Fig. 5(a) and 5(c) with Fig. 5(b) and 5(d), solution (6) with 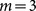 are more unstable than that with 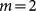.

**Figure 6:**
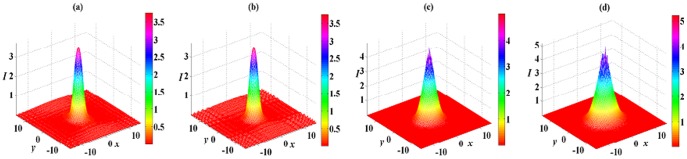
Initial value of solution (8) with 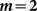 in the 2D extended 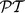-symmetric potential (7) at 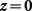 in (a). The numerical reruns of solution (8) with (b) 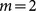, (c) 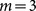 and (d) 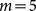 for DC and FP nonlinearities at 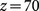. A 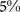 white noise are added to the initial values. All parameters are chosen as the same as those in Fig. 4.

Spatial soliton solution (8) also exhibits stable and unstable propagations in different nonlinear media. In the DC and FP nonlinear medium, solution (8) with 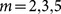 can stably propagate over tens of diffraction lengths. The influence of initial 5% white noise is suppressed, and only some small oscillations happen. Similar to the case shown in Fig. 4, the white noise only exerts an effect on the background of soliton and generates some small oscillations around the soliton for 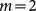 in Fig. 6(b). When 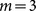 and 5, the white noise only affect the top part of soliton, and there are some small oscillations on the top part of soliton shown in Fig. 6(c) and 6(d). Compared Fig. 4(a) and 4(b) with Fig. 6(a) and 6(b), solution (6) is more stable than solution (8) for the case of 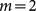 because white noise produce more small oscillations in Fig. 4(a) and 4(b) than that in Fig. 6(a) and 6(b).

**Figure 7:**
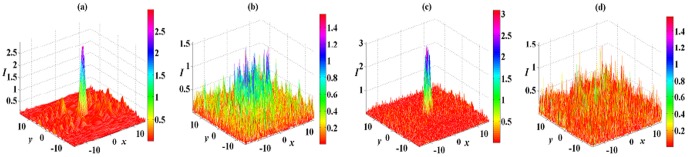
Unstable 2D spatial soliton solution (8) in the 2D extended 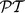-symmetric potential (7). The numerical reruns of solution (8) with (a),(c) 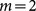, (b),(d) 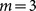 for (a), (b) DC and DP nonlinearities with 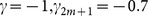 and (c), (d) FC and FP nonlinearities with 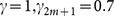 at 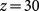. All other parameters are chosen as the same as those in Fig. 4.

Some examples of unstable spatial soliton solution (8) are presented in Fig. 7. In the DC and DP nonlinear medium and the FC and FP nonlinear medium, spatial solitons are broken down propagating after several diffraction lengths, and their original shapes can not be preserved, especially to the case of large nonlinear order 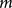. When 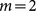 in Fig. 7(a) and 7(c), spatial solitons are distorted, then spread to the background, and next decay into noise. When 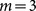 in Fig. 7(b) and 7(d), spatial solitons almost become noise. Therefore, solution (8) with 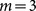 are more unstable than that with 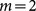, and this instability also adds with the increase of nonlinear order parameter 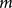.

## Conclusions

We analytically obtain two families of Gaussian-type soliton solutions of the (n+1)-dimensional Schrödinger equation with cubic and power-law nonlinearities in 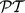-symmetric potentials. As an example, we discuss some dynamical behaviors of two dimensional soliton solutions. Their phase switches, powers and transverse power-flow densities are discussed. Results imply that the power flow and exchange from the gain toward the loss regions in the 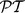 cell. Moreover, the linear stability analysis and the direct numerical simulation are carried out, which indicates that spatial Gaussian-type soliton solutions are stable below some thresholds for the imaginary part of 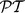-symmetric potentials in the defocusing cubic and focusing power-law nonlinear medium, while they are always unstable for all parameters in other media. These results will enrich the variety of higher dimensional structures in 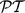-symmetric potential in the field of mathematical physics, and may also have potential values to the application of synthetic 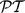-symmetric systems in nonlinear optics.
